# Instantaneous reproduction number and epidemic growth rate for predicting COVID-19 waves: the first 2 years of the pandemic in Spain

**DOI:** 10.3389/fpubh.2023.1233043

**Published:** 2023-09-15

**Authors:** Javier Llorca, Inés Gómez-Acebo, Jessica Alonso-Molero, Trinidad Dierssen-Sotos

**Affiliations:** ^1^Department of Preventive Medicine and Public Health, University of Cantabria, Santander, Spain; ^2^Consortium for Biomedical Research in Epidemiology and Public Health (CIBERESP), Institute of Health Carlos III, Madrid, Spain; ^3^Department of Preventive Medicine and Public Health, University of Cantabria-Instituto de Investigación Sanitaria de Valdecilla (IDIVAL), Santander, Spain

**Keywords:** COVID-19, SARS-CoV-2, reproductive number, epidemic growth rate, epidemic growth curve

## Abstract

**Introduction:**

Several indicators were employed to manage the COVID-19 pandemic. In this study, our objective was to compare the instantaneous reproductive number and the epidemic growth rate in the Spanish population.

**Methods:**

Data on daily numbers of cases, admissions into hospitals, admissions into ICUs, and deaths due to COVID-19 in Spain from March 2020 to March 2022 were obtained. The four “pandemic state indicators”, which are daily numbers of cases, admissions into hospitals, admissions into ICUs, and deaths due to COVID-19 in Spain from March 2020 to March 2022 were obtained from the Instituto de Salud Carlos III. The epidemic growth rate was estimated as the derivative of the natural logarithm of daily cases with respect to time. Both the reproductive number and the growth rate, as “pandemic trend indicators,” were evaluated according to their capacity to anticipate waves as “pandemic state indicators.”

**Results:**

Using both the reproductive number and the epidemic growth rate, we were able to anticipate most COVID-19 waves. In most waves, the more severe the presentation of COVID-19, the more effective the pandemic trend indicators would be.

**Conclusion:**

Besides daily number of cases or other measures of disease frequency, the epidemic growth rate and the reproductive number have different roles in measuring the trend of an epidemic. Naïve interpretations and the use of any indicator as a unique value to make decisions should be discouraged.

## 1. Introduction

During the COVID-19 pandemic, public health authorities dealt with the need to make decisions that affected not only the functioning of the health system but also people's activities. These decisions could include whether and when to order partial or total lockdown, disruption of economic life, closing of borders, and use of masks, among others, and when to resume normal activity. To make the above decisions, several pandemic indicators were used. An alert system should have at least two kinds of indicators (1): (1) a measure of state (i.e., what is the current burden of disease) and (2) a measure of trend (i.e., in what amount is the current burden of disease increasing or decreasing). Several state measures were available, namely the incidence of new cases in the whole population or specific age groups, the number of patients admitted to hospitals or ICUs, and the number of deaths. However, regarding the trend measures, the spectrum is rather limited, with the instantaneous reproductive number being the most widely used (2).

In brief, the basic reproductive number (R_0_) is the average number of secondary cases produced by each primary case at the beginning (i.e., time = 0) of the pandemic, when all people in the population are susceptible to the disease, and no control measures have been put in place. R_0_ depends on the number of contacts per day a primary case has, the probability of transmitting the disease to a contact, and the duration of infectiousness. Then, the instantaneous reproductive number at time *t* (R_t_) is the average number of secondary cases produced by each primary case at time t when some people are no longer susceptible or some control measures are enforced (2). Estimating R_t_ requires a model that at least should include the number of cases and the serial interval (i.e., the gap between a primary case with symptom onset and a secondary case with symptom onset) (3). Several methods of estimating R_t_ have been developed (4–6), including both dynamic and statistical approaches.

Dynamic models are usually variants of the Lotka-Euler equation (7). They require the estimation of parameters in a fully specified epidemiological model (say, SIR, SEIR, etc.) to analytically obtain R_t_. For instance, Arroyo-Marioli et al. (8) estimated R_t_ as a linear transformation of the growth rate in the context of a SIR model to study the evolution of COVID-19. Lipsitch et al. (5) derived R_t_ for a SEIR model as a non-linear transformation of the epidemic growth rate and applied it to the epidemic of SARS. Rodiah et al. (9) used an age-structured SEIRS model to study the evolution of R_t_ in Germany during the COVID-19 pandemic.

However, statistical models can also be used to study the characteristics of a pandemic based on time series data and information on the serial interval. For instance, let us suppose a disease with a fixed serial interval of 5 days. R_t_ is estimated by dividing the number of new cases on day *t* by the number of new cases on day *t-5*. Cori et al. (4) further developed this idea by considering the serial interval as a random variable with a known distribution, thus building a Bayesian estimator of R_t_. Furthermore, they developed the software EpiEstim, which helps estimate R_t_ from time-series data. Other authors, such as Wallinga and Teunis (6), also applied likelihood-based procedures to study the SARS epidemic.

The naïve interpretation of R_t_ is straightforward. Let us suppose a disease with a 10-day serial interval. R_t_ = 2 would mean that at day *t*+*10*, the number of new cases will be twice the number of new cases at day *t*. Therefore, R_t_ > 1 means the next generation of cases will outnumber the current generation (so the incidence is increasing), R_t_ < 1 means the opposite (so the incidence is decreasing), and R_t_ = 1 means the incidence is stable (6). This interpretation, which has been widely used in the media during the pandemic, has some caveats. First, R_t_ is an average estimate. Therefore, R_t_ in a region could be >1 while some parts of it have R_t_ < 1. Second, R_t_ is unitless, leading to its non-interpretability as a measure of time. For instance, let us suppose two diseases, A and B, with the same R_t_ = 2 but different serial intervals (10 days for disease A and 6 months for disease B). For disease A, R_t_ = 2 means that the number of new cases would double every 10 days, while for disease B, it means that the number of new cases would double every 6 months. Therefore, R_t_ says nothing about the speed at which the incidence is growing or declining (10). Finally, it has been pointed out that estimating R_t_ always requires a delay, as at least a serial interval would pass before the required information is gathered (10).

An alternative trend indicator is the epidemic growth rate, which has been much less used than R_t_ during the COVID-19 pandemic. The epidemic growth rate is the instantaneous change in incidence at time *t* and could be estimated as a derivative from a smoothed function of the incidence. As with R_t_, the naïve interpretation of the growth rate is uncomplicated, with 0 being the threshold: a growth rate > 0 means that the incidence is rising, and a growth rate < 0 means that the incidence is declining. Contrary to R_t_, the epidemic growth rate has units expressed as the change per time unit. For instance, a growth rate = 0.1/day means that the number of cases at day *t*+*1* will be 10% higher than at day *t*. A couple of advantages of the epidemic growth rate over the reproductive number are that the growth rate does not require model assumptions and that estimating it does not require waiting until the next generation of cases appears. Among its downsides, it has been noted that communicating a figure such as the epidemic growth rate, which is obtained as a derivative, is not as simple as communicating R_t_.

The purpose of this study is to compare the evolution of R_*t*_ and the epidemic growth rate in the Spanish population during the COVID-19 pandemic and to describe their respective abilities to anticipate an epidemic wave.

## 2. Methods

### 2.1. Notation

Instantaneous reproductive number: The average number of secondary cases produced by each primary case at time *t*. For simplicity, we have used “reproductive number” as a synonym. When abbreviating it, we have used R_t_ throughout the article.

Epidemic growth rate: The first derivative of a smoothed function of the number of cases at time *t*. For simplicity, we used “growth rate” as a synonym.

Generation time: The interval of time between successive infections in a transmission chain (11). We assume that the generation time is a random variable rather than a fixed value. The generation time is usually unobserved.

Serial time: The interval of time between the illness onsets of the infector and the infected (11). Serial time is generally and, in this article, used as a proxy for generation time. We assume that serial time is a random variable with a gamma distribution.

### 2.2. Data and sources of data

Data were obtained from the Instituto de Salud Carlos III (ISCIII) and proceeded from notified cases to the Spanish epidemiological surveillance net (Red Nacional de Vigilancia Epidemiológica). These data are publicly available at https://cnecovid.isciii.es/covid19/#documentaci%C3%B3n-y-dato (12) and were downloaded on 1 January 2023. Data include the number of new cases by date of diagnosis, new admissions to hospitals, new admissions to the ICU, and deaths. In the absence of the date of diagnosis, the date of declaration or date of symptom onset was used. Data were obtained on a daily basis from 1 March 2020 to 27 March 2022. Data collected after 27 March 2022 were excluded from the study as they only included people aged 60 years and above. Information on the prevalence of circulating variants was obtained from Our World in Data (13).

### 2.3. Identification of epidemic waves

Although there is no standard definition of the epidemic wave, both the media and public health authorities have identified a number of them. In this study, we used the periods identified for the evolution of the pandemic in Spain by the ISCIII as follows (14):

- First period: From the beginning of the pandemic until 21 June 2020, when the main restrictions on movement were removed (i.e., the end of the so-called “Estado de Alarma”).
- Second period: From 22 June 2020 to 6 December 2020.
- Third period: From 7 December 2020 to 14 March 2021.
- Fourth period: From 15 March 2021 to 19 June 2021.
- Fifth period: From 20 June 2021 to 13 October 2021.
- Sixth period: From 14 October 2021 to 27 March 2022.

### 2.4. Estimation of the instantaneous reproductive number

The effective reproductive number was estimated using the software EpiEstim 2 (15), which is accessible from the study of Thompson et al. (16). It uses a modification of Cori et al. (4) with a Bayesian framework where the *a priori* distribution of R_t_ is gamma with a mean of 5 and a standard deviation of 5, which makes the prior a little more informative. The serial interval distribution was set as having a gamma distribution with a mean of 5 days, a standard deviation of 1.9, and a maximum length of 10, as previously described with Spanish data (17). Then, EpiEstim 2 obtains the posterior distribution of R_t_ conditioned to the serial interval distribution by combining the R_t_ prior with the Poisson likelihood obtained from the data.

### 2.5. Estimation of the epidemic growth rate

The epidemic growth rate was estimated as the derivative of the natural logarithm of daily cases with respect to time. To make logarithms estimable, days with zero cases were set at 0.1. Then, a 5-day moving average was obtained. Finally, the moving average was fitted with a cubic spline, which was analytically differentiated. This procedure was carried out using the software Stata 16/SE (StataCorp, College Station, TX, USA).

## 3. Results

Although there is no one-to-one correspondence between waves and SARS-CoV-2 variants, some relationships could be noted. The first case of the alpha variant was identified at the end of 2020; the alpha variant share was 58% by 1 March 2021 and 85% by 29 March; then, it plateaued until 24 May 2021, after which the alpha variant share began to decline; thus, the emergence of the alpha variant corresponded to the third wave and its dominance to the fourth wave. The first case of delta variant in Spain was identified on 26 April 2021, reaching 19% by 21 June 2021, 53% by 5 July 2021, and 89% by 2 August 2021. Therefore, the emergence of the delta variant corresponded to the fourth wave and its dominance to the fifth wave. The first case of the omicron variant was identified on 29 November 2021; it reached 66% by 3 January 2022. Therefore, its emergence corresponded to the beginning of the sixth wave and its dominance to the second half of the sixth wave (Table 1). We limited our analysis to waves second to sixth, as the data in the first wave were much less reliable.

**Table 1:** Share of SARS-CoV-2 variants circulating in Spain every 4 weeks.

**Period (wave)**	**Date**	**Alpha**	**Delta**	**Omicron**
1	Any date	0	0	0
2	Any date	0	0	0
3	7 December 2020	0	0	0
3	4 January 2021	13	0	0
3	1 February 2021	28	0	0
3	1 March 2021	58	0	0
4	29 March 2021	85	0	0
4	26 April 2021	88	0.1	0
4	24 May 2021	86	2.8	0
5	21 June 2021	66	19	0
5	19 July 2021	16	75	0
5	16 August 2021	2	96	0
5	13 September 2021	0	100	0
5	11 October 2021	0.1	99	0
6	8 November 2021	0	100	0
6	6 December 2021	0	98	2
6	3 January 2022	0	34	66
6	31 January 2022	0	5	95
6	28 February 2022	0	0.3	99

Data are expressed in percentages. Source: Our World in Data (https://ourworldindata.org/covid-cases, accessed on 9 January 2023).

**Figure 1:**
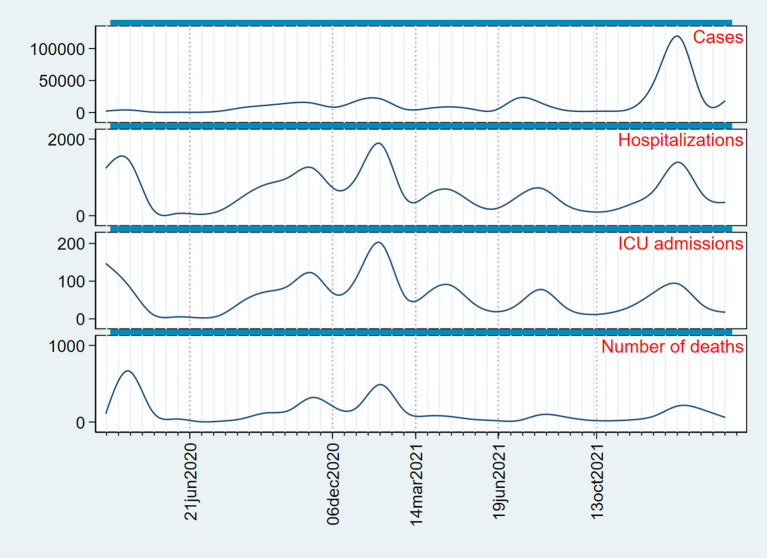
Daily number of cases, admissions into hospitals, admissions into the ICU, and deaths in Spain from 30 March 2020 to 27 March 2022 (smoothed with cubic splines). Vertical dotted lines (red) indicate the beginning and the ending of each wave. A grid has been added to serve as a reference; the distance between two consecutive lines in the grid is 2 weeks.

The daily number of cases, admissions into hospitals, admissions into the ICU, and deaths is summarized in Figure 1. All data in this figure has been smoothed with cubic splines to eliminate irregularities due to underreporting on weekends. The figure is arranged from the top to the bottom so that there is a gradient in severity. Vertical dotted lines (red) indicate the breaking points between waves. A grid has been drawn to serve as a temporal reference; the gap between two consecutive lines in the grid is 2 weeks. Let us consider the second wave (between 21 June 2020 and 6 December 2020). There is some delay in the maximum as we move from the top to the bottom of the figure, i.e., the more severe the disease, the later the wave peak; the total gap from the peak in number of cases to the peak in number of deaths is approximately 2 weeks. This total gap also occurred during waves third and fifth, the latter with a total gap of 4 weeks, but not during waves fourth and sixth, in which the peaks are nearly coincident (in the fourth wave, the peak in number of deaths is almost undetectable, but deaths occurred before any other peaks were achieved). A remarkable finding is that, except for the fifth wave, there were no more than 2 weeks between the peak in number of cases and the peak in number of deaths. Therefore, there was little time to take public health measures between the increase in cases and the increase in more severe disease or death, making it necessary to use epidemic indicators other than the number of cases.

**Figure 2:**
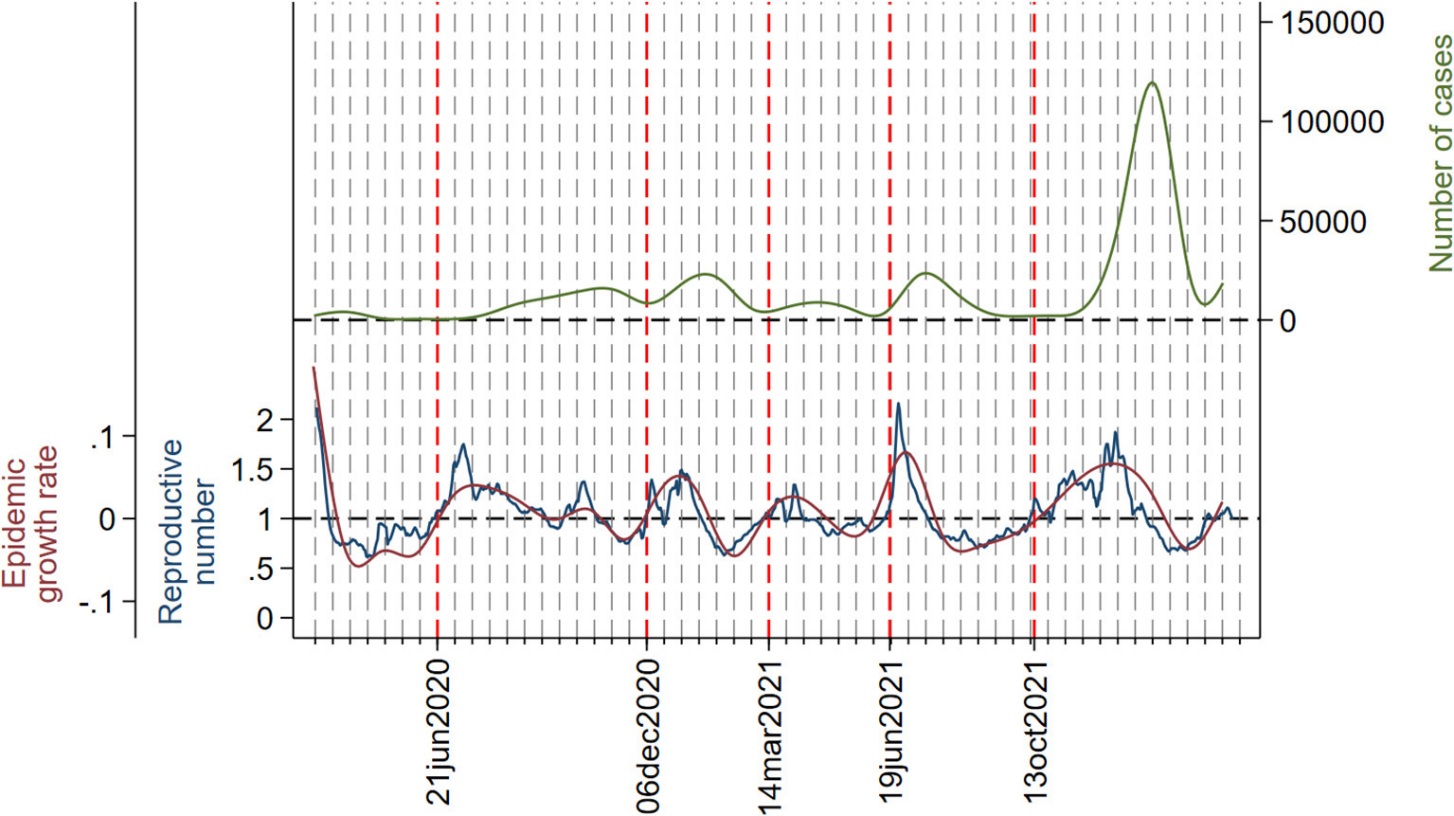
Daily number of cases (smoothed with cubic splines) (green line), instantaneous reproductive number (blue line), and epidemic growth rate (red line). Dotted vertical lines (red) and the corresponding dates in the x-axis indicate the beginning and the ending of each wave. A grid has been added to serve as a reference; the distance between two consecutive lines in the grid is 2 weeks. The y-axis on the left is set so that epidemic growth rate = 0 and reproductive number = 1 coincide.

The evolution of the daily number of cases, instantaneous reproductive number, and epidemic growth rate are displayed in Figure 2. In each wave from the second to the sixth, both the reproductive number and the growth rate peaked widely before the number of cases. The second wave is somehow peculiar, meaning that the number of cases increased gradually so that both the reproductive number and the growth rate peaked twice: the first one, which is the highest, at approximately 15 weeks before the summit of cases, and the second one at 3 weeks before the peak in number of cases. In the third, fourth, fifth, and sixth waves, both the reproductive number and the growth rate peaked 4 weeks before the number of cases. In all waves, peaks in reproductive number and growth rate coincided with each other.

**Figure 3:**
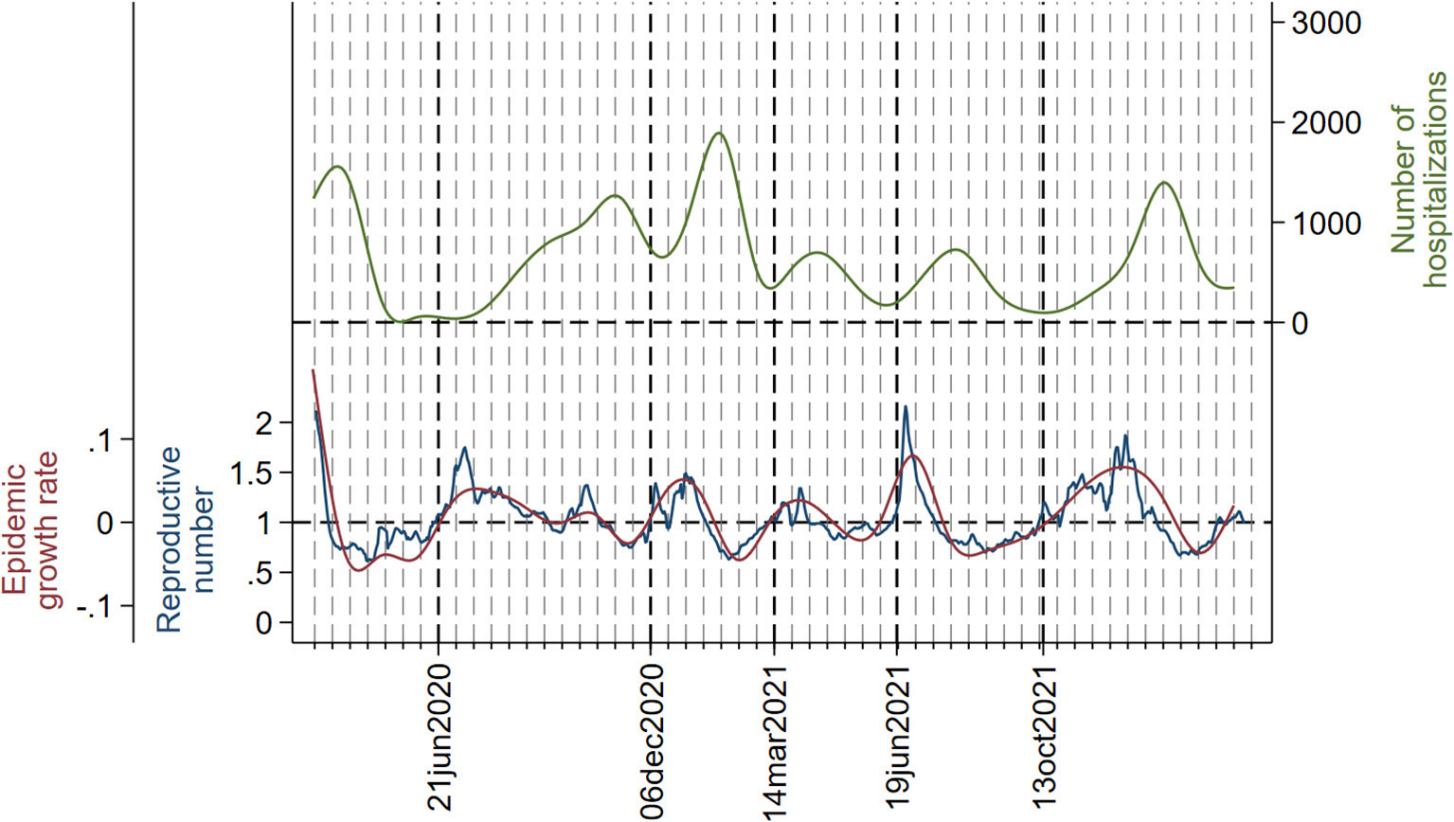
Daily number of admissions into hospitals (smoothed with cubic splines) (green line), instantaneous reproductive number (blue line), and epidemic growth rate (red line). Dotted vertical lines (red) and the corresponding dates in the x-axis indicate the beginning and the ending of each wave. A grid has been added to serve as a reference; the distance between two consecutive lines in the grid is 2 weeks. The y-axis on the left is set so that epidemic growth rate = 0 and reproductive number = 1 coincide.

**Figure 4:**
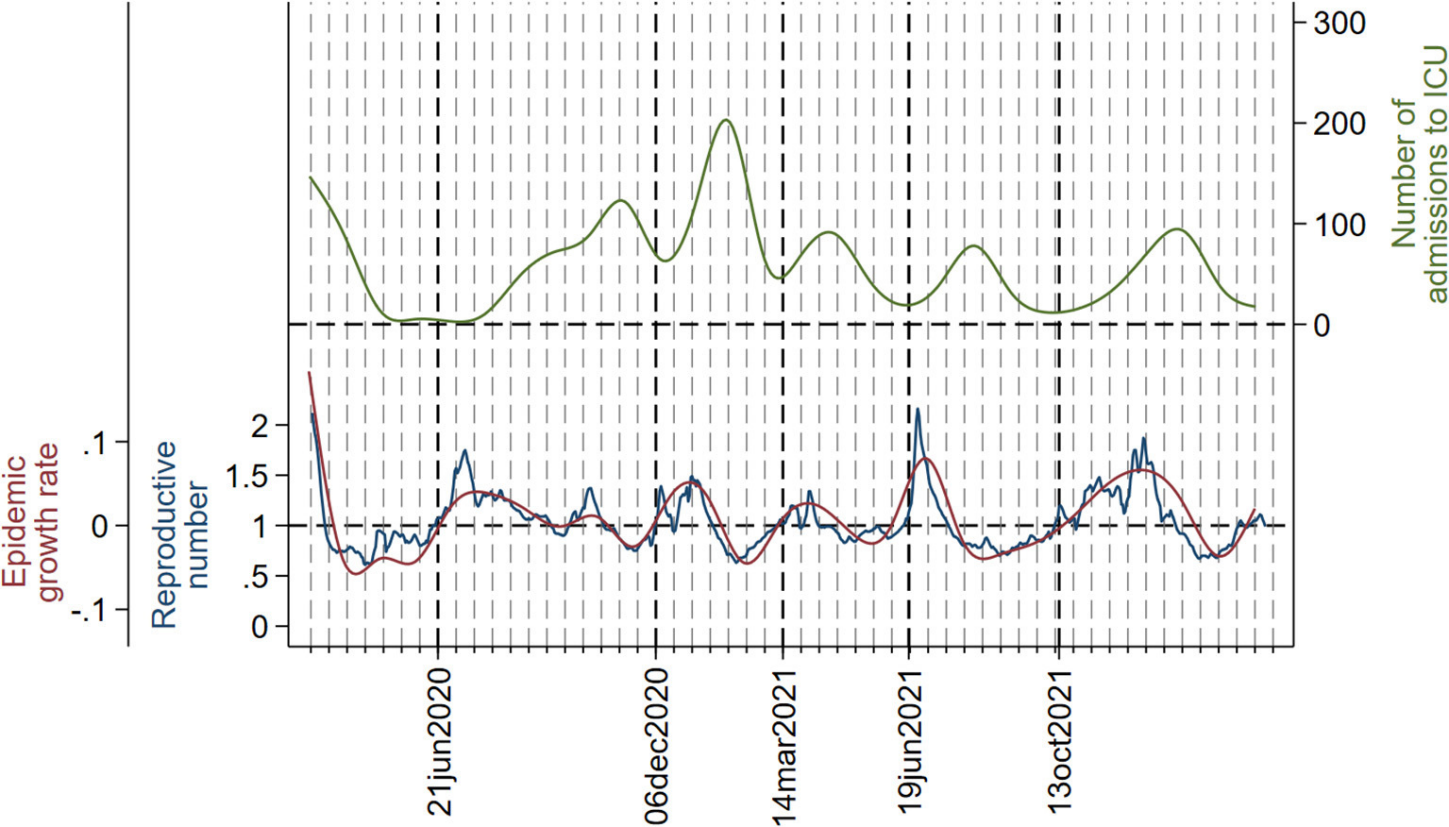
Daily number of admissions to the ICU (smoothed with cubic splines) (green line), instantaneous reproductive number (blue line), and epidemic growth rate (red line). Dotted vertical lines (red) and the corresponding dates in x-axis indicate the beginning and the ending of each wave. A grid has been added to serve as reference; distance between two consecutive lines in the grid is 2 weeks. The y-axis on the left is set so that epidemic growth rate = 0 and reproductive number = 1 coincide.

**Figure 5:**
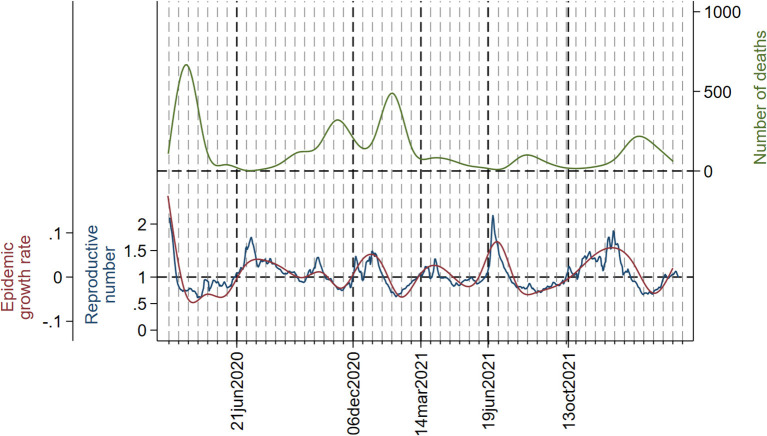
Daily number of deaths (smoothed with cubic splines) (green line), instantaneous reproductive number (blue line), and epidemic growth rate (red line). Dotted vertical lines (red) and the corresponding dates in x-axis indicate the beginning and the ending of each wave. A grid has been added to serve as reference; the distance between two consecutive lines in the grid is 2 weeks. The y-axis on the left is set so that epidemic growth rate = 0 and reproductive number = 1 coincide.

Figures 3–5 show the joint evolution of reproductive number and growth rate with the number of admissions into hospitals (Figure 3), admissions into the ICU (Figure 4), and deaths (Figure 5). In all three figures, there was a notable temporal gap between the summits of reproductive number and growth rate and those of the disease indicators, which happened in each wave. The only exception was the small peak in number of deaths (Figure 5) in the fourth wave, which happened early in the wave and almost coincided with peaks in reproductive number and growth rate.

To further demonstrate the relationships between the new cases or deaths and both the reproductive number and the epidemic growth rate, we applied the same methods to the pandemic evolution in Italy, the first European country hit by COVID-19, and the United Kingdom. The results are reported in the Supplementary material. Both the growth rate and the reproductive number increased some weeks before any wave in incidence cases or deaths. It is remarkable that, in Italian data, increases in reproductive numbers in July 2021 (about the time the delta variant emerged) and December 2021/January 2022 (related to the first omicron-variant wave) were more abrupt than those in growth rate. The Supplementary material also displays the uncertainty in estimating the reproductive number.

## 4. Discussion

The main result of this study is that both the instantaneous reproductive number and the epidemic growth rate anticipated the waves of severe or deadly COVID-19 several weeks before they could be anticipated by the number of cases. This result was consistent for admissions to the hospitals, admissions to the ICU, and deaths, as well as for most waves in the first 2 years of the pandemic. A public health implication would be that real-time estimation of either the reproductive number or the growth rate would allow the authorities to make decisions faster than waiting for the evolution of the number of cases.

The evolution of reproductive numbers and epidemic growth rates mirror each other, which is not surprising as both are transformations from the number of cases curve, provided that the generation time does not change. With this assumption in mind, reproductive numbers and epidemic growth rates encode the same information (7). Therefore, when deciding which indicator could be more useful, we should focus not on the information it provides but on its estimation properties, as discussed in the following paragraphs.

First, let us zoom in on the characteristics shared by the reproductive number and the growth rate. (A) Both the reproductive number and the epidemic growth rates are averages across different sub-populations (e.g., age groups or geographical units), making it possible for an estimate to hide great disparities in transmission between, say, people aged 60–64 vs. people aged 10–14 or between rural vs. urban areas. (B) They are both averages over time, forcing the researcher or the public health authority to decide the time window for estimating them. A brief time window may yield few points for a reliable estimation, whereas an extended time window could compromise the ability to capture the immediate characteristics of trend indicators (10). (C) Any trend indicator requires data to be reliably gathered during the studied period. The reliability of data collection during the pandemic has been affected by multiple factors. These include shifts in clinical and epidemiological testing criteria, variations in test quality—ranging from lab-intensive methods in the early stages to at-home rapid tests introduced approximately a year later—and fluctuations in the volume of tests conducted. Additional complications arise from changes in reporting guidelines, inconsistencies in data definitions, and delays in reporting.

Second, let us focus on differences when estimating the reproductive number and the growth rate. The main difference in estimating them is that the reproductive number requires a model (18), whereas the growth rate is model-free (10). Different models to estimate the reproductive number coexist (4–6), some using a dynamic model such as the SIR-type (5) and others using a statistical model (4, 6). They all need at least some competence to estimate (10) or assume (18) the generation time or serial time distribution. It is noteworthy that serial intervals could change during an epidemic due to public health measures, behavioral changes (19), or the emergence of new viral variants. Therefore, the reproductive number estimates are susceptible to the chosen model, so different assumptions could eventually lead to different estimates (19, 20), making it less practical to make decisions in an epidemic. For instance, Challen et al. (19) have shown the way that changes in serial interval assumptions could affect the reproduction number estimates: assuming larger means would lead to reproductive number estimates away from the critical value 1, and this effect is more important in the dynamic phases of the epidemic (i.e., when ascending or descending, not in the peak), while assuming a larger standard deviation has little effect in the ascending or descending phases but could lead to an estimate that the reproductive number would cross 1 later than estimates obtained with a lower standard deviation.

Even after setting the model and its assumptions, real-time estimation of the reproductive number is not straightforward. First, cases could be reported some days after the beginning of symptoms, making the estimation of the reproductive number misleading. Second, estimating the instantaneous reproductive number for day *t* requires information on the immediate next generation of cases, which could last for a complete serial interval to be completed. For instance, in our study, we used a serial interval with a mean = 5 days, standard deviation = 1.9, and a maximum of 10 days. Therefore, to have the complete set of necessary data available, the reproductive number could not have been estimable until day *t*+*10* (i.e., *t* + maximum of the serial interval). Some ways of reducing this gap have been published. Some models assume a fixed value for serial intervals (for instance, 5 days for all secondary cases), eventually sacrificing accuracy for immediacy. In this regard, Wallinga et al. (7) have shown that reproductive number estimates under such an assumption are biased upward so that they put an upper bound on the values the reproductive number can take. Another approach is to treat the beginning of symptoms as a missing data problem. In this regard, De Salazar et al. (17) first imputed the day of the beginning of symptoms conditionally to the day of the report and then imputed the day of the beginning of symptoms for those cases not yet reported. The whole procedure allowed them to estimate the reproductive number in near real-time. However, their procedure had only been tested in March–April 2020, when most diagnosed cases were symptomatic and many were severe. Notably, the wide availability of testing was not on the agenda until several months later. Therefore, the method by De Salazar et al. requires further validation in a setting where most diagnosed cases are asymptomatic.

In this regard, the main advantage of the epidemic growth rate is that its calculation does not require data on the next generation of cases (10), which allows its earlier estimation. However, as mentioned above, estimating the growth rate from noisy data requires some smoothing procedures, which might be equivalent to modeling assumptions (18). Therefore, although Pellis et al. (10) favored the growth rate based on the lack of assumptions it requires, Parag et al. (18) considered that both the growth rate and reproductive number are valuable, the first being better to indicate the speed at which incidence is growing or declining and the second being more intuitive for public communication.

The analysis performed in this article depends on the reported data. Therefore, our results could be sensitive to underreporting, which varies widely from country to country. For instance, the incidence-detection ratio has been estimated to be as low as 6.9% globally but 44.6% in high-income countries (21). Regarding Spanish data, the incidence-detection ratio was estimated at 45.3%, with important differences from region to region, ranging from 29.3% in the Canary Islands to 61.9% in La Rioja (21). Not only did the underreporting vary geographically but also throughout the development of the pandemic. Thus, the infection-detection ratio trends continued to increase (21), which could be associated with the wider availability of diagnostic tests. The impact of underreporting cases in our analysis is difficult to evaluate. We consider three scenarios: Scenario (1): Underreporting was a constant in the pandemic: Had this been the case, underreporting would have affected the results only as a matter of scale (i.e., the actual number of cases would have been obtained by multiplying the reported numbers by a constant), leaving the reproduction number and growth rate unchanged. We could hardly consider this scenario reliable, as the wider availability of diagnostic tests could eventually lead to lower underreporting. Scenario (2): Underreporting could decrease from wave to wave, remaining almost constant during each wave. In this scenario, estimates of R_t_ and growth rate during each wave would have still been stable, eventually allowing their use to forecast the wave evolution and make decisions. We consider this scenario to be a fair approximation as test availability did not change abruptly; for instance, the infection-detection ratio in Figure 2 shown in Barber et al. (21) remains constant but slow increases as the pandemic continued. Scenario (3): Underreporting significantly decreased during any wave. If this were the case, the reproductive number and the growth rate would have been downscaled as the wave went on. Under this scenario, the utility of the reproductive number and the growth rate would have been questionable unless reliable estimates of underreporting were available in real time.

In conclusion, despite limitations imposed by data quality, the epidemic growth rate and the reproductive number would have different roles in measuring the trend of an epidemic together with measures of disease frequency. Naïve interpretations and the use of any indicator as a unique value to make decisions should be discouraged.

## Data availability statement

Publicly available datasets were analyzed in this study. This data can be found here: https://cnecovid.isciii.es/covid19/#documentaci%C3%B3n-y-datos.

## Author contributions

JL and IG-A have contributed to the conception and design of the study. IG-A and TD-S have directed the implementation of the study, including quality assurance and control. The first draft of the manuscript was written by JL, IG-A, and JA-M. JL and TD-S have supervised and designed the study's analytic strategy, reviewing, and preparing the Methods and Results sections. IG-A and JA-M have prepared and written the Introduction and the Discussion sections of the text. All authors have read and approved the final manuscript.

## References

[1] Brooks-Pollock E Read JM McLean AR Keeling MJ Danon L. Mapping social distancing measures to the reproduction number for COVID-19. Philos Trans R Soc B. (2021) 376:276. 10.1098/rstb.2020.0276 34053268 PMC8165600

[2] Jewell NP Lewnard JA. On the use of the reproduction number for SARS-COV-2: estimation, misinterpretations and relationships with other ecological measures. J R Stat Soc Ser A Stat Soc. (2022) 185:S16–27. 10.1111/rssa.12860 35942193 PMC9350332

[3] Giesecke J. Modern Infectious Disease Epidemiology. Third eds. London: CRC Press. (2017).

[4] Cori A Ferguson NM Fraser C Cauchemez S. A new framework and software to estimate time-varying reproduction numbers during epidemics. Am J Epidemiol. (2013) 178:1505–12. 10.1093/aje/kwt133 24043437 PMC3816335

[5] Lipsitch M Cohen T Cooper B Robins JM Ma S James L. Transmission dynamics and control of severe acute respiratory syndrome. Science. (2003) 300:1966–1970. 10.1126/science.1086616 12766207 PMC2760158

[6] Wallinga J Teunis P. Different epidemic curves for severe acute respiratory syndrome reveal similar impacts of control measures. Am J Epidemiol. (2004) 160:509–16. 10.1093/aje/kwh255 15353409 PMC7110200

[7] Wallinga J Lipsitch M. How generation intervals shape the relationship between growth rates and reproductive numbers. Proc R Soc B Biol Sci. (2007) 274:599–604. 10.1098/rspb.2006.3754 17476782 PMC1766383

[8] Arroyo-Marioli F Bullano F Kucinskas S Rondón-Moreno C. Tracking R of COVID-19: A new real-time estimation using the Kalman filter. PLoS ONE. (2021) 16:e0244474. 10.1371/journal.pone.0244474 33439880 PMC7806155

[9] Rodiah I Vanella P Kuhlmann A Jaeger VK Harries M Krause G. Age-specific contribution of contacts to transmission of SARS-CoV-2 in Germany. Eur J Epidemiol. (2023) 38:39–58. 10.1007/s10654-022-00938-6 36593336 PMC9807433

[10] Pellis L Birrell PJ Blake J Overton CE Scarabel F Stage HB. Estimation of reproduction numbers in real time: conceptual and statistical challenges. J R Stat Soc Ser A Stat Soc. (2022) 185:S112–30. 10.1111/rssa.12955 37063605 PMC10100071

[11] Chen D Lau YC Xu XK Wang L Du Z Tsang TK. Inferring time-varying generation time, serial interval, and incubation period distributions for COVID-19. Nat Commun. (2022) 13:7727. 10.1038/s41467-022-35496-8 36513688 PMC9747081

[12] RENAVE. Incidencias acumuladas y curvas epidémicas. Available online at: https://cnecovid.isciii.es/covid19/#documentaci%C3%B3n-y-datos (accessed January 1, 2023).

[13] Mathieu E Ritchie H Rodés-Guirao L Appel C Gavrilov D Giattino C. Coronavirus (COVID-19) Cases. Available online at: https://ourworldindata.org/covid-cases (accessed January 9, 2023).

[14] Equipo COVID-19, RENAVE, CNE, CNM (ISCIII). Situación de COVID-19 en España a 2 de diciembre de 2022. (2022). Available online at: https://www.isciii.es/QueHacemos/Servicios/VigilanciaSaludPublicaRENAVE/EnfermedadesTransmisibles/Documents/INFORMES/Informes%20COVID-19/INFORMES%20COVID-19%202022/Informe%20n%C2%BA%20157%20Situaci%C3%B3n%20actual%20de%20COVID-19%20en%20Espa%C3%B1a%20a%202%20de%20diciembre%20de%202022.pdf (accessed February 26, 2023).

[15] Thompson RN Stockwin JE van Gaalen RD Polonsky JA Kamvar ZN Demarsh PA. Improved inference of time-varying reproduction numbers during infectious disease outbreaks. Epidemics. (2019) 29:100356. 10.1016/j.epidem.2019.100356 31624039 PMC7105007

[16] Thompson R Stockwin J van Gaalen R Polonsky J Kamvar Z Demarsh P. EpiEstim App. Available online at: https://shiny.dide.imperial.ac.uk/epiestim (accessed January 7, 2023).

[17] De Salazar PM Lu F Hay JA Gómez-Barroso D Fernández-Navarro P Martínez E V. Near real-time surveillance of the SARS-CoV-2 epidemic with incomplete data. PLoS Comput Biol. (2022) 18:e1009964. 10.1371/journal.pcbi.1009964 35358171 PMC9004750

[18] Parag K V Thompson RN Donnelly CA. Are Epidemic Growth Rates More Informative than Reproduction Numbers? J R Stat Soc Ser A Stat Soc. (2022) 185:S5–S15. 10.1111/rssa.12867 35942192 PMC9347870

[19] Challen R Brooks-Pollock E Tsaneva-Atanasova K Danon L. Meta-analysis of the severe acute respiratory syndrome coronavirus 2 serial intervals and the impact of parameter uncertainty on the coronavirus disease 2019 reproduction number. Stat Methods Med Res. (2022) 31:1686–703. 10.1177/09622802211065159 34931917 PMC9465543

[20] Parag K V Donnelly CA. Using information theory to optimise epidemic models for real-time prediction and estimation. PLoS Comput Biol. (2020) 16:e1007990. 10.1371/journal.pcbi.1007990 32609732 PMC7360089

[21] Barber RM Sorensen RJD Pigott DM Bisignano C Carter A Amlag JO. Estimating global, regional, and national daily and cumulative infections with SARS-CoV-2 through 14 November, 2021: a statistical analysis. Lancet. (2022) 399:2351–80. 10.1016/S0140-6736(22)00484-6 35405084 PMC8993157

