# Supplementary material for: Instantaneous reproduction number and epidemic growth rate for predicting COVID-19 waves: the first 2 years of the pandemic in Spain
Source: Front Public Health. 2023 Sep 15;11:1233043. doi: 10.3389/fpubh.2023.1233043 (PMC10540620; doi:10.3389/fpubh.2023.1233043)

**Instantaneous reproduction number and epidemic growth rate for predicting COVID-19 waves: The first two years of the pandemic in Spain**

**Javier Llorca^1,2^, Inés Gómez-Acebo^2,3^, Jessica Alonso-Molero^2,3^, Trinidad Dierssen-Sotos^2,3^**

^1^Retired Professor. University of Cantabria, Santander, Spain
^2^Consortium for Biomedical Research in Epidemiology and Public Health (CIBERESP), Institute of Health Carlos III, Madrid, Spain
^3^University of Cantabria-IDIVAL, Santander, Spain

APPENDIX

**Supplementary analysis of the COVID-19 pandemic in Italy and the United Kingdom and uncertainty of R_t_ estimations**

In this appendix we present the instantaneous reproduction number and epidemic growth rate obtained in Italy and the United Kingdom. The methods of estimating the instantaneous reproduction number and the epidemic growth rate are the same already described in the article.

1. The pandemic in Italy

Data on daily new cases and deaths have been obtained from <https://covid19.who.int/WHO-COVID-19-global-data.csv> (consulted August 16^th^, 2023).

Period of study: March 1^st^, 2020 to August 8^th^, 2023.

Supplementary figure 1. Daily number of new cases (smoothed by cubic splines) (green line), instantaneous reproductive number (blue line) and epidemic growth rate (red line) in Italy, March 2020 to August 2023. Vertical dashed grid has been added as reference; consecutive lines in the grid are separated by 2 weeks.


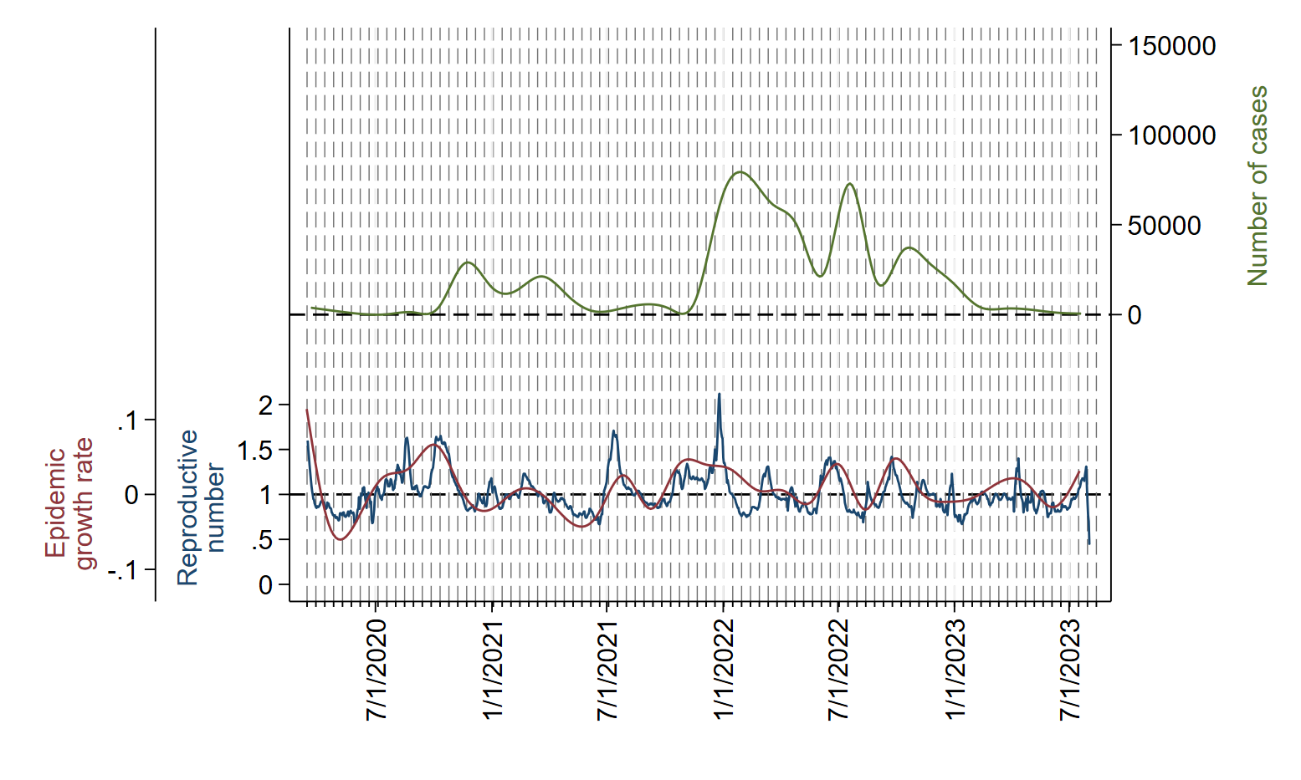


Supplementary figure 2. Daily number of deaths (smoothed by cubic splines) (green line), instantaneous reproductive number (blue line) and epidemic growth rate (red line) in Italy, March 2020 to August 2023. Vertical dashed grid has been added as reference; consecutive lines in the grid are separated by 2 weeks.


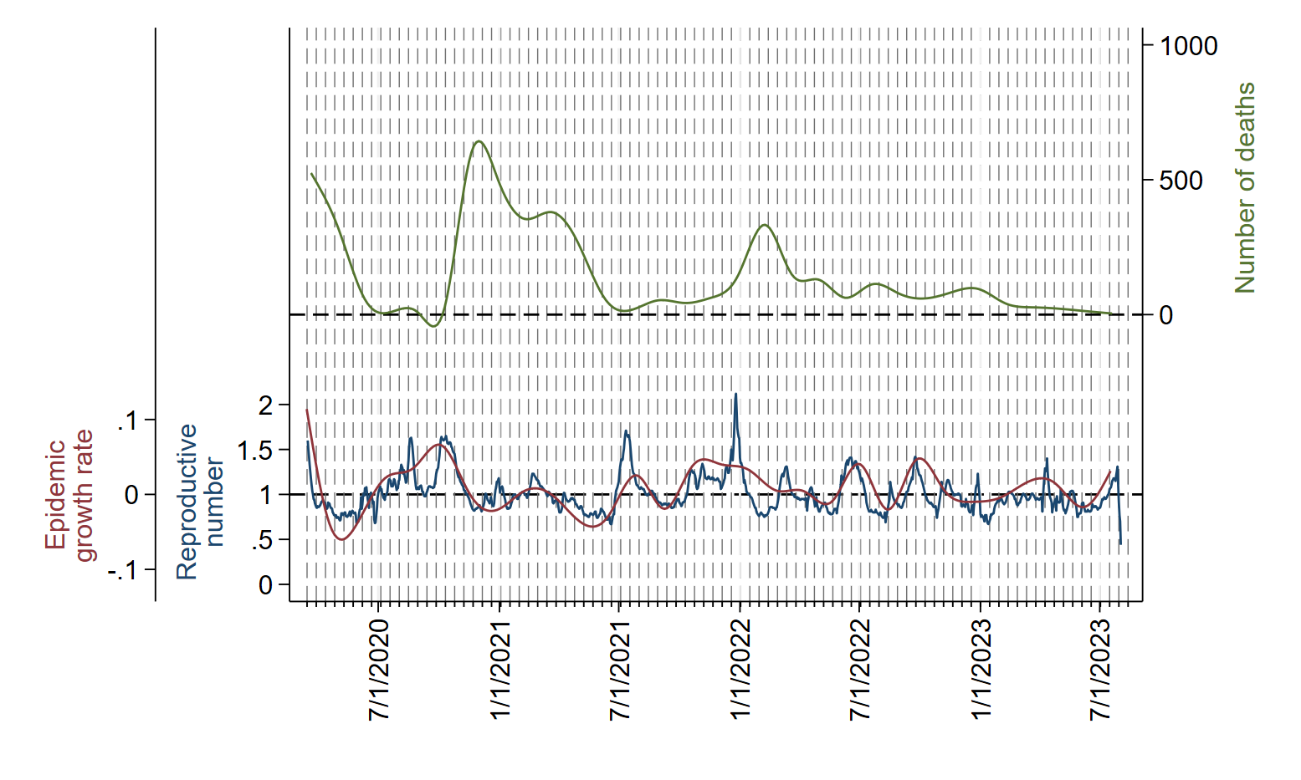


1. The pandemic in the United Kingdom

Data on daily new cases and deaths have been obtained from <https://covid19.who.int/WHO-COVID-19-global-data.csv> (consulted August 16th, 2023).

Period of study: March 1st, 2020 to August 8th, 2023.

Supplementary figure 3. Daily number of new cases (smoothed by cubic splines) (green line), instantaneous reproductive number (blue line) and epidemic growth rate (red line) in the United Kingdom, March 2020 to August 2023. Vertical dashed grid has been added as reference; consecutive lines in the grid are separated by 2 weeks.


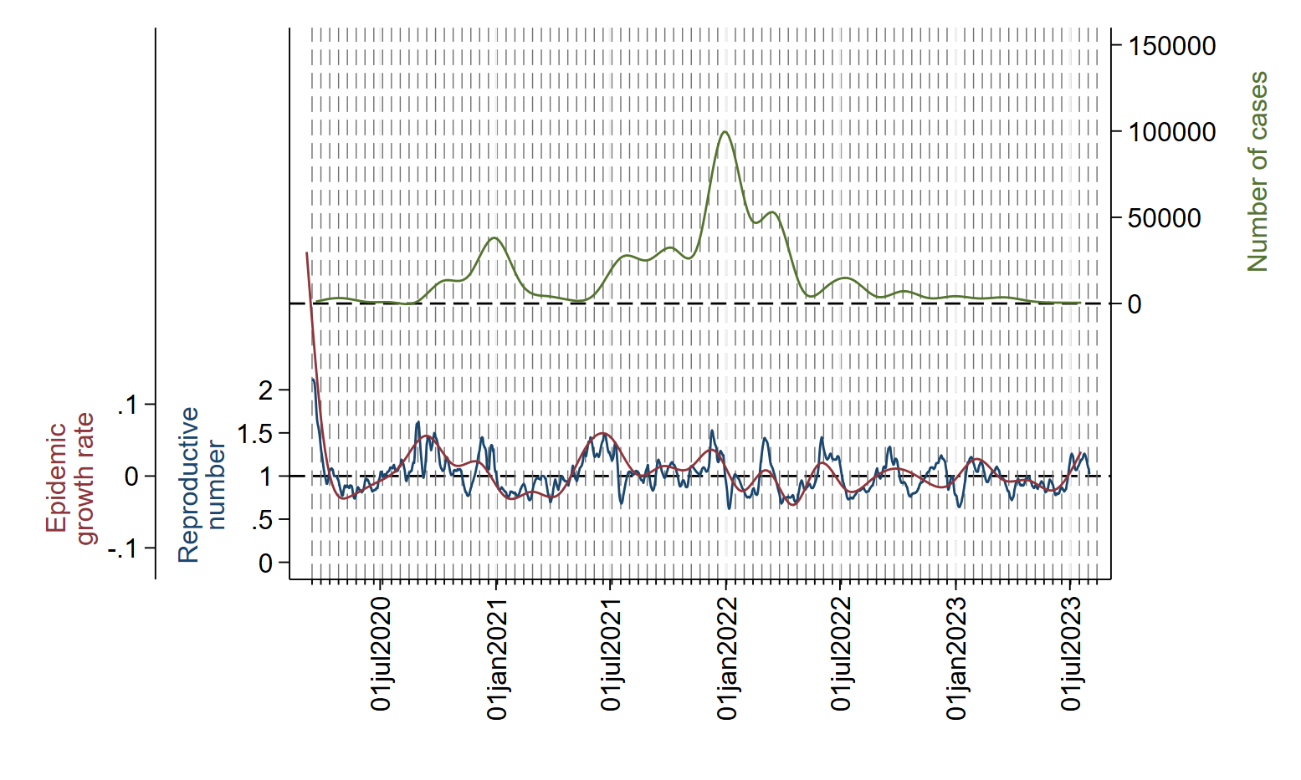


Supplementary figure 4. Daily number of deaths (smoothed by cubic splines) (green line), instantaneous reproductive number (blue line) and epidemic growth rate (red line) in the United Kingdom, March 2020 to August 2023. Vertical dashed grid has been added as reference; consecutive lines in the grid are separated by 2 weeks.

3.
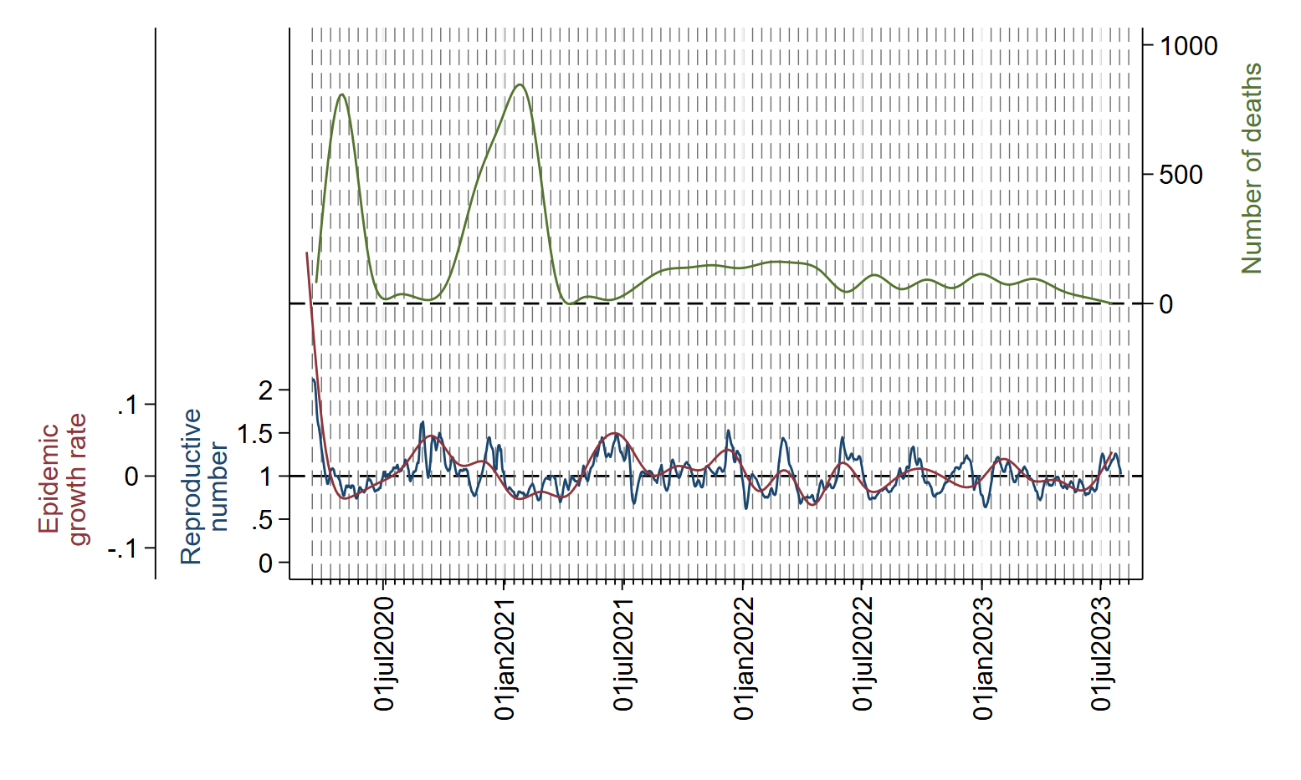


3. Uncertainty in the instantaneous reproductive number

Supplementary Figure 5. Median estimation of the instantaneous reproductive number (blue line) and its 95% credible interval (light blue area), as estimated with EpiEstim 2. Spain, March 2020 – March 2022. The epidemic growth rate (red line) is included as reference.


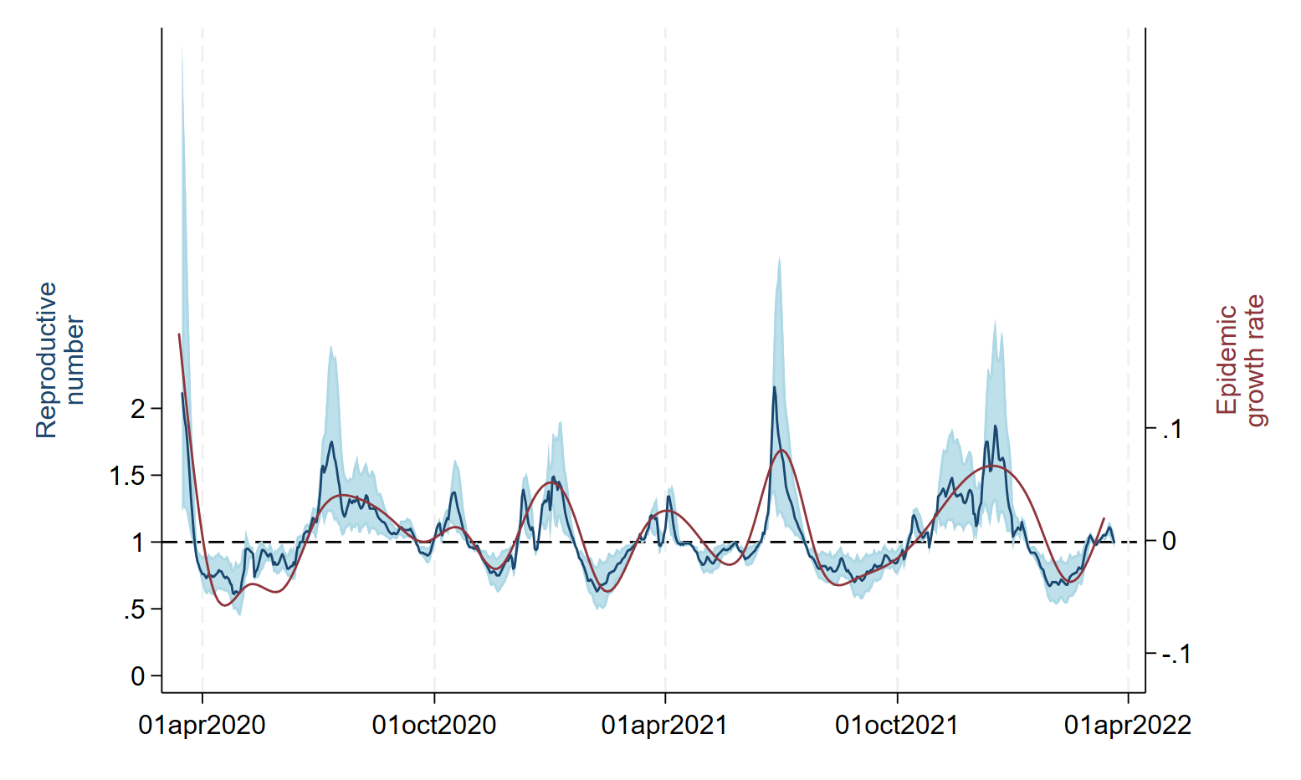

Supplement: Supplementary file 1 [file Data_Sheet_1.DOCX]
